# Supplementary material for: Mapping molecular assemblies with fluorescence microscopy and object-based spatial statistics
Source: Nat Commun. 2018 Feb 15;9:698. doi: 10.1038/s41467-018-03053-x (PMC5814551; doi:10.1038/s41467-018-03053-x)
Supplement: Supplementary file 1 — Supplementary Information [file 41467_2018_3053_MOESM1_ESM.pdf]

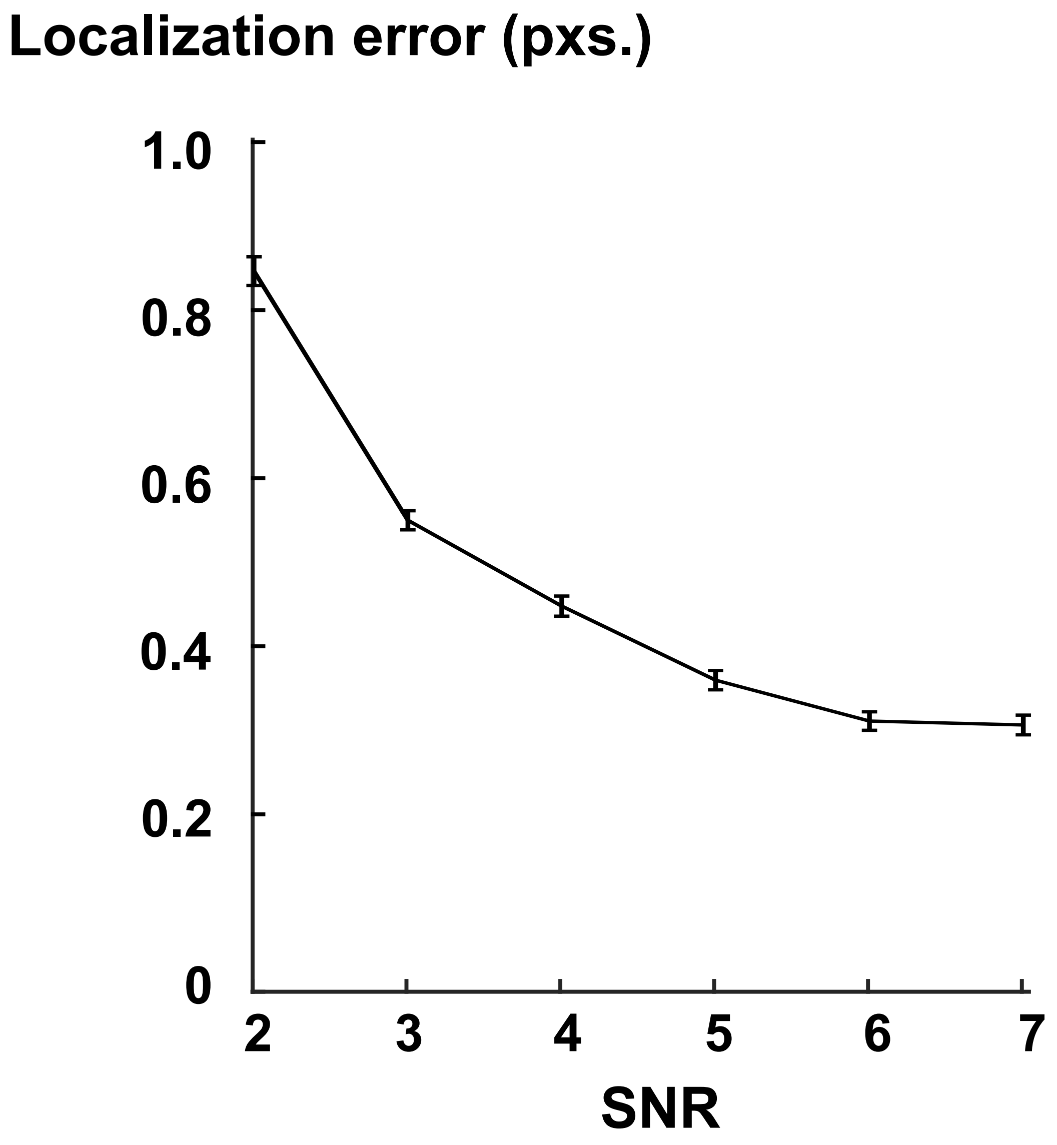

Supplementary Figure 1 : Spot's localization error as function of image SNR (error bars = s.e.m., 10 synthetic images with n=100 spots per condition).

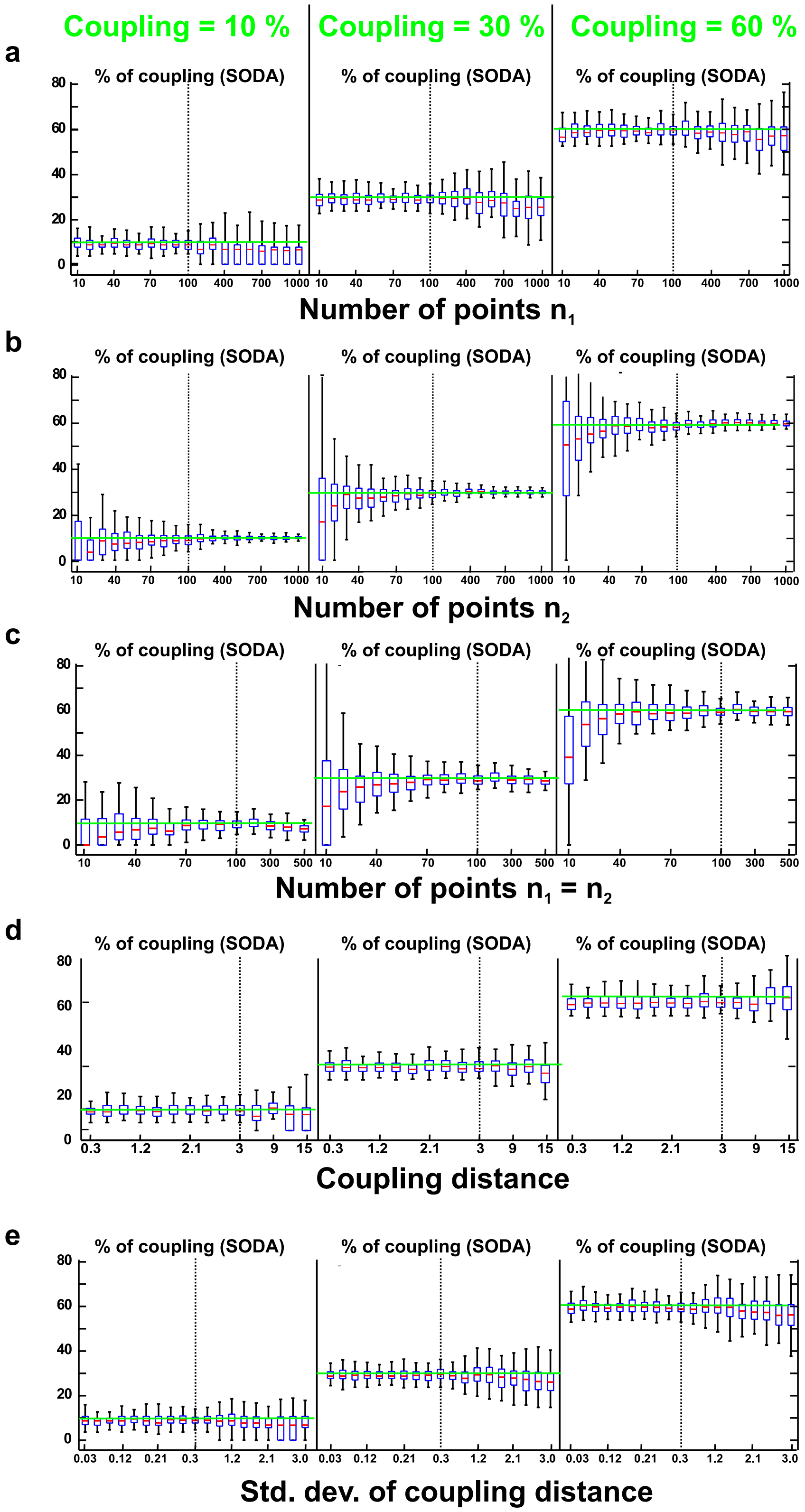

Supplementary Figure 2 : Testing the sensitivity of SODA to several parameters with point-process simulations. SODA is challenged using Thomas point process simulations with a wide range of coupling parameters. We use three different coupling percentages (10, 30 and 60 %) and change the following parameters: **a-** the number n_1 of points in point-process A_1, **b-** the number n_2 of points in point-process A_2, **c-** numbers $n_1=n_2$ together, **d-** the coupling distance and **e-** the standard deviation of the coupling distance. Box plots are represented for the different values of the parameters (n=1000 Monte-Carlo simulations per condition).

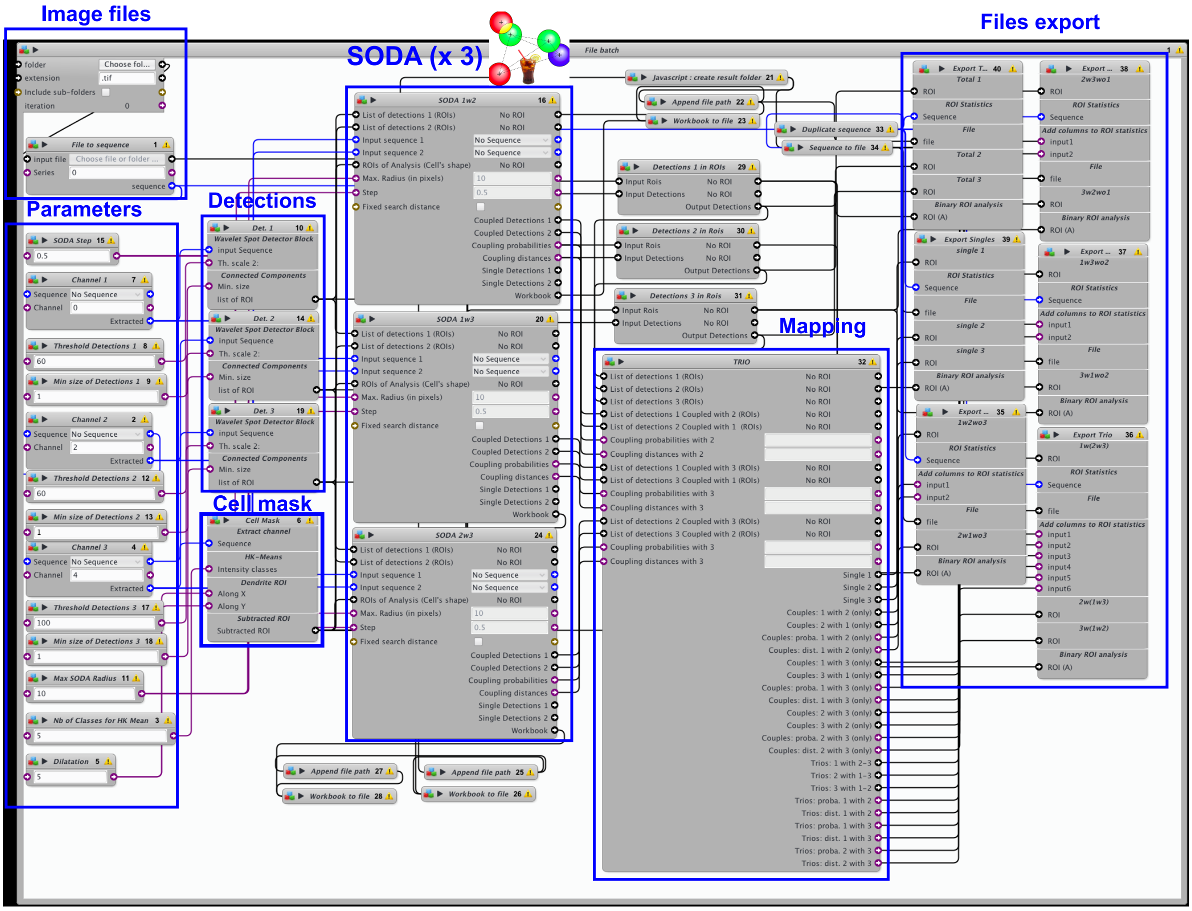

Supplementary Figure 3 : Screenshot of the protocol developed in Icy to analyze automatically multiple three-color SIM images of hippocampal neurons. First, user defines the location of image files and parameters of the analysis such as the different molecule channels in images, the thresholds for automatic segmentation of spots and cell mask, or the maximum radius of SODA analysis. Then a series of blocks perform the detection of Homer, PSD95 and Synapsin spots, and delineate the neuronal mask automatically. Three SODA blocks then use the detection localizations and the cell mask to statistically map the positions of isolated and coupled spots and compute coupling probabilities and p-values. Statistical color-maps of molecular assemblies at a population level, as well as morphological and spatial parameters of each molecular assembly, are then exported in different files and can be used for robust, quantitative analysis of molecular assemblies.

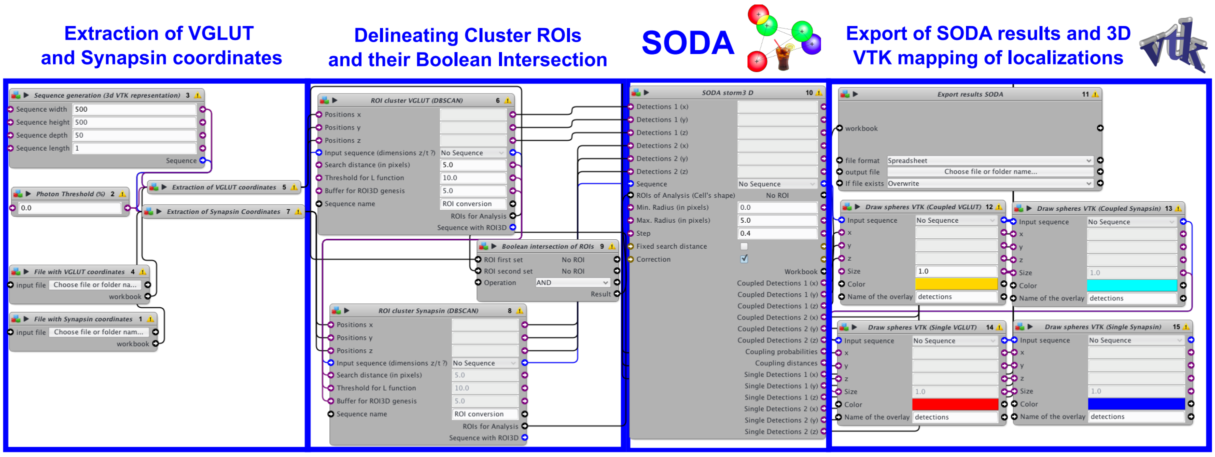

Supplementary Figure 4 : Screenshot of the protocol developed in Icy to analyze automatically three-dimensional STORM images of VGLUT and Synapsin localizations. A first series of blocks extract the (x,y,z) coordinates of localizations from microscope's files. Then, blocks using density-based clustering (DBSCAN) automatically delineate ROIs around volumes where VGLUT and Synapsin localizations are clustered. The intersection between VGLUT and Synapsin ROIs is an input parameter of the SODA STORM 3D block, as well as localizations' coordinates and other parameters of SODA such as the minimal and maximal radii of analysis. Finally, a last series of blocks export the results of the coupling analysis in user-specified files, and color-map single and coupled 3d localizations with VTK in Icy.

Supplementary Methods

Computation of the standard deviation σ of G

To account for the boundaries of the field of view, and the possible underestimation of each component $G_i = G(r_i, r_{i+1})$ of the Ripley's vector **G**, we use the standard Ripley's boundary correction [1]

$$k(x,y) = \frac{\text{Volume}\{C(x, d(x,y)\}}{\text{Volume}\{C(x, d(x,y) \cap \text{ROI}\}} \quad (1)$$

which is inversely proportional to the proportion of the circle (sphere in 3D) $C(x, d(x,y))$ centered on x with radius $d(x,y)$ that is included in the ROI. By denoting

$$g_0(x, y, r_i, r_{i+1}) = \mathbf{1}_{\{r_i \le d(x,y) \le r_{i+1}\}} k(x,y) - \frac{\mu_i}{\text{Volume}\{\text{ROI}\}} \quad (2)$$

with $\boldsymbol{\mu} = [\mu_i]_{0 \le i \le N-1}$ the mean of **G** under the null hypothesis of (red) objects 2 randomness, we have

$$\sigma_i^2 = \frac{\text{Volume}\{\text{ROI}\}^2}{n_1^2 n_2^2} \text{E}\left\{\left(\sum_{x \in A_1} \sum_{y \in A_2} g_0(x, y, r_i, r_{i+1})\right)^2\right\} \quad (3)$$

with n_1 and n_2 the number of objects in point processes A_1 and A_2, and E the expected mean with respect to A_2 random distribution. We decompose σ_i^2 as

$$\begin{aligned} \sigma_i^2 = \frac{\text{Volume}\{\text{ROI}\}^2}{n_1^2 n_2^2} \Bigg(& \sum_{x_1 \in A_1} \sum_{y_1 \in A_2} \text{E}\{g_0(x_1, y_1, r_i, r_{i+1})\} \\ & + \sum_{x_1 \neq x_2 \in A_1} \sum_{y_1 \in A_2} \text{E}\{g_0(x_1, y_1, r_i, r_{i+1}) g_0(x_2, y_1, r_i, r_{i+1})\} \\ & + \sum_{x_1 \in A_1} \sum_{y_1 \neq y_2 \in A_2} \text{E}\{g_0(x_1, y_1, r_i, r_{i+1}) g_0(x_1, y_2, r_i, r_{i+1})\} \\ & + \sum_{x_1 \neq x_2 \in A_1} \sum_{y_1 \neq y_2 \in A_2} \text{E}\{g_0(x_1, y_1, r_i, r_{i+1}) g_0(x_2, y_2, r_i, r_{i+1})\} \Bigg). \quad (4) \end{aligned}$$

Under the null hypothesis, A_2 is an homogeneous Poisson process (complete spatial randomness (CSR)) and we rewrite equation (3) as

$$\begin{aligned} \sigma_i^2 = \frac{\text{Volume}\{\text{ROI}\}^2}{n_1^2 n_2^2} \Bigg(& \sum_{x_1 \in A_1} \int_{\text{ROI}} g_0^2(x_1, y, r_i, r_{i+1}) dy + \sum_{x_1 \neq x_2 \in A_1} \int_{\text{ROI}} g_0(x_1, y, r_i, r_{i+1}) g_0(x_2, y, r_i, r_{i+1}) dy \\ & + \sum_{x_1 \in A_1} \frac{n_2 - 1}{\text{Volume}\{\text{ROI}\}} \left(\int_{\text{ROI}} g_0(x_1, y, r_i, r_{i+1}) dy \right)^2 \\ & + \sum_{x_1 \neq x_2 \in A_1} \frac{n_2 - 1}{\text{Volume}\{\text{ROI}\}} \int_{\text{ROI}} g_0(x_1, y, r_i, r_{i+1}) dy \int_{\text{ROI}} g_0(x_2, y, r_i, r_{i+1}) dy \Bigg) \quad (5) \end{aligned}$$

Because $\int_{ROI} g_0(x, y, r_i, r_{i+1}) dy = 0$ for any $x_1 \in A_1$, Eq. (5) reduces to

$$\sigma_i^2 = \frac{\text{Volume}\{\text{ROI}\}}{n_1^2 n_2}\left(\sum_{x_1 \in A_1} \int_{\text{ROI}} g_0^2(x_1, y, r_i, r_{i+1})dy + \sum_{x_1 \neq x_2 \in A_1} \int_{\text{ROI}} g_0(x_1, y, r_i, r_{i+1}) g_0(x_2, y, r_i, r_{i+1})dy\right) \quad (6)$$

Consequently, we are left with the computation of the integrals

$$\tilde{I}_1(x_1, r_i, r_{i+1}) = \int_{ROI} g_0^2(x_1, y, r_i, r_{i+1})dy \quad (7)$$

and

$$\tilde{I}_2(x_1, x_2, r_i, r_{i+1}) = \int_{ROI} g_0(x_1, y, r_i, r_{i+1}) g_0(x_2, y, r_i, r_{i+1})dy \quad (8)$$

Denoting $g\,(x_1, y, r_i, r_{i+1}) = \mathbf{1}_{\{r_i \leq d(x,y) \leq r_{i+1}\}} k(x, y)$, we first expand $\tilde{I}_1(x_1, r_i, r_{i+1})$

$$\tilde{I}_1(x_1, r_i, r_{i+1}) = \int_{ROI} \left(g(x_1, y, r_i, r_{i+1}) - \frac{\mu_i}{\text{Volume}\{\text{ROI}\}}\right)^2 dy$$
$$= \int_{ROI} g^2(x_1, y, r_i, r_{i+1})dy - \frac{\mu_i^2}{\text{Volume}\{\text{ROI}\}} \quad (9)$$

We observe that for points $x_1 \in A_1$ that are at a distance $d(x_1, \partial[\text{ROI}]) > r_{i+1}$ from the domain boundary $\partial[\text{ROI}]$, there is no need for boundary correction (i.e. $k(x, y) = 1$). Thus, we can decompose

$$\int_{ROI} g^2(x_1, y, r_i, r_{i+1})dy = \mathbf{1}_{\{d(x_1, \partial[ROI]) > r_{i+1}\}} \mu_i + \mathbf{1}_{\{d(x_1, \partial[ROI]) < r_{i+1}\}} \int_{ROI} g^2(x_1, y, r_i, r_{i+1})dy \quad (10)$$

Denoting

$$A_h = \{y \in \text{ROI}, \text{such that } r_i \leq d(x_1, y) \leq r_{i+1} \text{ and given that } d(x_1, \partial[ROI]) = h < r_{i+1}\} \quad (11)$$

we rewrite Eq. (10) as

$$\int_{ROI} g^2(x_1, y, r_i, r_{i+1})dy = \mathbf{1}_{\{d(x_1, \partial[ROI]) > r_{i+1}\}} \mu_i + \mathbf{1}_{\{d(x_1, \partial[ROI]) = h < r_{i+1}\}} \int_{A_h} k^2(x_1, y)dy \quad (12)$$

In two dimensions, assuming that the boundary is locally linear (i.e. that the boundary is smooth, with a local curvature radius much larger than maximum searching distance r_N), the boundary correction is given by [1]

$$k(x_1, y) = \left(1 - \frac{1}{\pi}\cos^{-1}\left(\frac{\min(h, d(x_1, y))}{d(x_1, y)}\right)\right)^{-1} \quad (13)$$

There is no analytical expression for $\int_{A_h} k^2(h, y)dy$ and we thus used Monte-Carlo numerical integrations : For given $0 < h < r$, we used $n_y = 1000$ random prositions $y_i, 1 \leq i \leq n$ in A_h and approximate

$$\int_{A_h} k^2(h, y)dy \approx \text{Volume}\{A_h\} \frac{1}{n_y} \sum_{i=1}^{n_y} k^2(x_1, y_i) \quad (14)$$

In three dimensions, the boundary correction is given by

$$k(x_1, y) = \frac{2d(x_1, y)}{d(x_1, y) + \min\,(h, d(x_1, y))} \quad (15)$$

and in that case, we can compute the closed form expression

$$\int_{A_h} k^2(h, y)dy = \int_{r_i}^{\min(h, r_i)} 4\pi u^2 du + \int_{\min(h, r_i)}^{\text{r}_{i+1}} \left(\frac{2u}{u + h}\right)^2 4\pi u^2 du \quad (16)$$

that is

$$\int_{A_h} k^2(h,y)dy = \frac{4}{3}\pi\left(r_{i+1}^3 F\left(\frac{h}{r_{i+1}}\right) - r_i^3 F\left(\frac{\min(h,r_i)}{r_i}\right)\right) \qquad (17)$$

with $F(\alpha) = 2 - 3\alpha + 6\alpha^2 - 4\alpha^3 + 6\alpha^3(\alpha\log(2\alpha) - \log(1+\alpha))$.
Finally, we have computed that

$$\tilde{I}_1(x_1, r_i, r_{i+1}) = \mathbf{1}_{\{d(x_1,\partial[ROI])>r_{i+1}\}}\mu_i + \mathbf{1}_{\{d(x_1,\partial[ROI])=h\le r_{i+1}\}}\int_{A_h} k^2(h,y)dy - \frac{\mu_i^2}{\text{Volume}\{\text{ROI}\}} \qquad (18)$$

where $\int_{A_h} k^2(h,y)dy$ is either given by Monte-Carlo simulations in two dimensions (Eq. (14)) or by the closed form expression (17) in three dimensions.
Concerning $\tilde{I}_2$, we first expand it as

$$\tilde{I}_2(x_1, x_2, r_i, r_{i+1}) = \int_{ROI} g(x_1, y, r_i, r_{i+1})g(x_2, y, r_i, r_{i+1})dy - \frac{\mu_i^2}{\text{Volume}\{\text{ROI}\}} \qquad (19)$$

Neglecting cross-edge effects ($k(x_1, x_2) \approx 1$), we have

$$\tilde{I}_2(x_1, x_2, r_i, r_{i+1}) = \int_{ROI} \mathbf{1}_{\{r_i\le d(x_1,y)\le r_{i+1}\}}\mathbf{1}_{\{r_i\le d(x_2,y)\le r_{i+1}\}}dy - \frac{\mu_i^2}{\text{Volume}\{\text{ROI}\}} \qquad (20)$$

which is equal to

$$\tilde{I}_2(x_1, x_2, r_i, r_{i+1}) = \text{Volume}\{Ring(x_1, r_i, r_{i+1}) \cap Ring(x_2, r_i, r_{i+1})\} - \frac{\mu_i^2}{\text{Volume}\{\text{ROI}\}} \qquad (21)$$

where $Ring(x_1, r_i, r_{i+1}) \cap Ring(x_2, r_i, r_{i+1})$ is the boolean intersection between rings centered at x_1 and x_2 in ROI, with internal radius r_i and external radius r_{i+1}. We can decompose this intersection as function of disk intersections $D(x_1, r_i) \cap D(x_2, r_i)$, $D(x_1, r_{i+1}) \cap D(x_2, r_{i+1})$ and $D(x_1, r_i) \cap D(x_2, r_{i+1})$ (or balls intersections $B(x_1, r_i) \cap B(x_2, r_i)$, $B(x_1, r_{i+1}) \cap B(x_2, r_{i+1})$ and $B(x_1, r_i) \cap B(x_2, r_{i+1})$ in three dimensions) :

$$\begin{aligned}&\text{Volume}\{Ring(x_1, r_i, r_{i+1}) \cap Ring(x_2, r_i, r_{i+1})\}\\ &\quad = \text{Volume}\{D(x_1, r_i) \cap D(x_2, r_i)\} + \text{Volume}\{D(x_1, r_{i+1}) \cap D(x_2, r_{i+1})\}\\ &\quad - 2\text{Volume}\{D(x_1, r_i) \cap D(x_2, r_{i+1})\} \qquad (22)\end{aligned}$$

In two dimensions, the area of disks' intersection Volume$\{D(x_1, r_i) \cap D(x_2, r_{i+1})\}$ is equal to

$$\begin{aligned}&\text{Volume}\{D(x_1, r_i) \cap D(x_2, r_{i+1})\}\\ &\quad = \mathbf{1}_{\{d(x_1,x_2)\le r_i+r_{i+1}\}}\Bigg[r_i^2\cos^{-1}\left(\frac{d(x_1,x_2)^2 + r_i^2 - r_{i+1}^2}{2d(x_1,x_2)r_i}\right)\\ &\quad + r_{i+1}^2\cos^{-1}\left(\frac{d(x_1,x_2)^2 + r_{i+1}^2 - r_i^2}{2d(x_1,x_2)r_{i+1}}\right)\\ &\quad + \frac{1}{2}\left((d(x_1,x_2)^2 - (r_i + r_{i+1})^2)(d(x_1,x_2)^2 - (r_{i+1} - r_i)^2)\right)^{\frac{1}{2}}\Bigg] \qquad (23)\end{aligned}$$

Which reduces for $r_i = r_{i+1} = r$ to

$$\begin{aligned}&\text{Volume}\{D(x_1, r) \cap D(x_2, r)\}\\ &\quad = \mathbf{1}_{\{d(x_1,x_2)\le 2r\}}\left[2r^2\cos^{-1}\left(\frac{d(x_1,x_2)}{2r}\right) - \frac{d(x_1,x_2)}{2}(4r^2 - d(x_1,x_2)^2)^{\frac{1}{2}}\right] \qquad (24)\end{aligned}$$

In three dimensions, the volume of balls'interssection is equal to

$$\text{Volume}\{B(x_1, r_i) \cap B(x_2, r_{i+1})\} = \frac{\pi \mathbf{1}_{\{d(x_1,x_2) \le r_i + r_{i+1}\}}}{12 d(x_1, x_2)} \Big[(d(x_1, x_2)^2 + 2d(x_1, x_2)(r_i + r_{i+1}) - 3(r_i - r_{i+1})^2)\big(r_i + r_{i+1} - d(x_1, x_2)\big)^2 \Big] \quad (25)$$

And reduces for $r_i = r_{i+1} = r$ to

$$\text{Volume}\{B(x_1, r_i) \cap B(x_2, r_{i+1})\} = \frac{\pi \mathbf{1}_{\{d(x_1,x_2) \le 2r\}}}{12} \Big[(d(x_1, x_2) \; + 4r)\big(2r - d(x_1, x_2)\big)^2 \Big] \quad (26)$$

Finally, reinjecting the final expression of $\tilde{I}_1$ (Eq. (18)) and $\tilde{I}_2$ (Eq. (21)) in the variance equation (Eq. (6)), we obtain that

$$\sigma_i^2 = \frac{\text{Volume}\{\text{ROI}\}}{n_1^2 n_2} \left(\sum_{x_1 \in A_1} \mathbf{1}_{\{d(x_1, \partial[ROI]) > r_{i+1}\}} \mu_i + \mathbf{1}_{\{d(x_1, \partial[ROI]) = h < r_{i+1}\}} \gamma_{h,2} + \sum_{x_1 \ne x_2 \in A_1} \text{Volume}\{Ring(x_1, r_i, r_{i+1}) \cap Ring(x_2, r_i, r_{i+1})\} \right) - \frac{\mu_i^2}{n_2}, \quad (27)$$

that we rewrite

$$\sigma_i^2 = \frac{\text{Volume}\{\text{ROI}\}}{n_1^2 n_2} \left(\sum_{x_1 \in A_1} \mu_i \left(1 + b(x_1, r_i, r_{i+1}) - \frac{n_1 \mu_i}{\text{Volume}\{\text{ROI}\}} \right) + \sum_{x_1 \ne x_2 \in A_1} \text{Volume}\{Ring(x_1, r_i, r_{i+1}) \cap Ring(x_2, r_i, r_{i+1})\} \right), \quad (28)$$

where the volume (area in two dimensions) of the rings'intersection $\text{Volume}\{Ring(x_1, r_i, r_{i+1}) \cap Ring(x_2, r_i, r_{i+1})\}$ is given by Eq. (22), and $b(x_1, r_i, r_{i+1}) \ge 0$ is a boundary correction term for $x_1 \in \mathrm{A}_1$, such that $d(x_1, \partial[ROI]) < r_{i+1}$,

$$b(x_1, r_i, r_{i+1}) = \mathbf{1}_{\{d(x_1, \partial[ROI]) = h < r_{i+1}\}} \left(\int_{A_h} k^2(h, y) dy - \mu_i \right). \quad (29)$$

Supplementary References

[1] Getis, A. and Franklin, J. (1987). Second-order neighborhood analysis of mapped point patterns. Ecology, 68:473-477.
