# Supplementary material for: Mapping molecular assemblies with fluorescence microscopy and object-based spatial statistics
Source: Nat Commun. 2018 Feb 15;9:698. doi: 10.1038/s41467-018-03053-x (PMC5814551; doi:10.1038/s41467-018-03053-x)
Supplement: Supplementary file 3 — Description of Additional Supplementary Files [file 41467_2018_3053_MOESM3_ESM.pdf]

Description of Additional Supplementary Files

File Name: Supplementary Movie 1
Description: Imaging of two pre-synaptic molecules (Synapsin and VGLUT) with the 3D-STORM Vutara system, statistical analysis of the coupling between single localisations with SODA, and 3D rendering of the statistical map of coupling with VTK in Icy.

File Name: Supplementary Movie 2
Description: Tutorial for Icy installation.

File Name: Supplementary Movie 3
Description: Tutorial for using the SIM protocol in Icy.

File Name: Supplementary Movie 4
Description: Tutorial for using the STORM protocol in Icy.
